# Detection of prostate cancer by an FDG-PET cancer screening program: results from a Japanese nationwide survey

**Published:** 2014

**Authors:** Ryogo Minamimoto, Michio Senda, Seishi Jinnouchi, Takashi Terauchi, Tomio Inoue

**Affiliations:** 1 Division of Nuclear Medicine, Department of Radiology, National Center for Global Health and Medicine, Tokyo, Japan; 2 Division of Molecular Imaging, Institute of Biomedical Research and Innovation, Kobe, Japan; 3 Atsuchi Memorial Institute of Radiology, Atsuchi Memorial Clinic PET Center, Kagoshima, Japan; 4 Division of Diagnostic Radiology, National Cancer Center Hospital, Tokyo, Japan; 5 Department of Radiology, Yokohama City University, Graduate School of Medicine, Yokohama, Japan

**Keywords:** Cancer screening, FDG-PET, PET/CT, Prostate cancer, PSA

## Abstract

**Objective(s):**

The aim of this study was to analyze detection rates and effectiveness of ^18^F-fluorodeoxyglucose positron emission tomography (FDG-PET) cancer screening program for prostate cancer in Japan, which is defined as a cancer-screening program for subjects without known cancer. It contains FDG-PET aimed at detection of cancer at an early stage with or without additional screening tests such as prostate-specific antigen (PSA) and magnetic resonance imaging (MRI).

**Methods:**

A total of 92,255 asymptomatic men underwent the FDG-PET cancer screening program. Of these, 504 cases with findings of possible prostate cancer in any screening method were analyzed.

**Results:**

Of the 504 cases, 165 were verified as having prostate cancer. Of these, only 61 cases were detected by FDG-PET, which result in 37.0% relative sensitivity and 32.8% positive predictive value (PPV). The sensitivity of PET/computed tomography (CT) scanner was higher than that of dedicated PET (44.0% vs. 20.4%). However, the sensitivity of FDG-PET was lower than that of PSA and pelvic MRI. FDG-PET did not contribute to improving the sensitivity and PPV when performed as combined screening.

**Conclusion:**

PSA should be included in FDG-PET cancer screening programs to screen for prostate cancer.

## Introduction

Prostate cancer has the fourth-highest age-adjusted incidence and the sixth-highest mortality rate among all cancers in Japan and is predicted to increase in the future (1, 2). The sensitivity of prostate-specific antigen (PSA) for screening prostate cancer scored 80-82% with a cut-off level of 4.0 ng/mL (3-5), which is much higher than for digital rectal examination (DRE) (4) and transrectal ultrasound (TURS) (3,5). After the introduction of PSA, the mortality rate of prostate cancer in United States of America reached a peak at 1990-1992 and decreased to 36% of the 1990s in 2005 (6). According to a study in Tyrol, Austria, implementation of PSA testing was associated with a reduction in the prostate cancer mortality rate (7, 8), but the argument continues over the effect of PSA testing on the prostate cancer mortality reduction.

The result of ^18^F-fluorodeoxyglucose positron emission tomography (FDG-PET) cancer screening in Japan between 2006 and 2009 was reported for a total of 155,456 healthy subjects, detecting prostate cancer in 165 of a total of 1,912 cancer cases (9), and it was the 4th most common cancer found in this program. The aim of the present study was to analyze prostate cancers found by FDG-PET cancer screening program.

## Methods

### Subjects

An overview of the survey of the 155,456 subjects who underwent FDG-PET cancer screening has already been reported. Subjects underwent FDG-PET cancer screening in a total of 233 facilities (2006, 59; 2007, 57; 2008, 58; and 2009, 59) (9). This study focused on the results of possible prostate cancer cases, duplicating part of the available data. This study involved 92, 255 men who underwent FDG-PET cancer screening. All study protocols in this retrospective, observational study were approved by the institutional review board. The Japanese Society of Nuclear Medicine and the Clinical PET Promoting Committee published ‘‘The Guidelines of FDG-PET Cancer Screening’’ in 2004 and revised it in 2007, with the aim of improving the quality of the FDG-PET cancer screening program (10, 11). All facilities performing the FDG-PET cancer screening program followed these guidelines. This guideline mainly states the limitation of FDG-PET for detection of prostate cancer, however it also suggests that the case with focal FDG uptake in prostate has possibility of prostate cancer.

### Contents of the investigation

The survey required the following information for all possible prostate cancer cases: age group; occurrence of annual FDG-PET cancer screening; FDG-PET (including FDG-PET/computed tomography [CT]) image findings; results of combined cancer screening tests such as PSA and pelvic magnetic resonance imaging (MRI) if any test was done; and final results obtained by further examinations. Criteria for positive values of PSA depended on the individual facility. The final result was classified as three categories: “proven cancer”; “cancer excluded”; and “strict follow-up because cancer was not ruled out”. If the case was “proven cancer”, the survey required the definitive diagnostic procedure for prostate cancer; and staging according to Union for International Cancer Control (UICC) clinical stage (sixth version). The final staging of prostate cancer was based on the results of clinical staging and/or pathological staging.

### Definition of terms

The term “FDG-PET” is defined as an examination performed by a dedicated PET or a PET/CT scanner. An “FDG-PET cancer screening program” is defined as a cancer-screening program for subjects without known cancer that contains FDG-PET aimed at detection of cancer at an early stage with or without additional screening tests such as PSA and MRI. The information obtained from the CT integrated in the PET/CT scanner was regarded as part of the PET/CT imaging. This definition was adopted because the CT findings could not be ignored when interpreting the PET/CT imaging.

### Statistical analysis

The chi-square test for independence was performed to compare sensitivities among findings from PET/CT, FDG-PET, and other screening tests. Because subjects with negative findings in the FDG-PET screening program did not undergo further screening, we computed “relative sensitivity”, which is the rate of proven cancer detected by a certain test out of those detected by any test. Also positive predictive value (PPV) was calculated for the test. A value of *P*<0.05 was considered significant.

## Results

Prostate cancer was suspected in 504 of the 92,255 subjects. Of the 504 subjects, 165 cases of prostate cancer (PPV, 32.7%) and 131 cases of benign disease were found, and 84 subjects underwent strict follow-up. The final diagnosis of cancer was confirmed by surgical procedures (21 cases) and prostate biopsy (124 cases), and was not mentioned in 20 cases. Prostate cancer was most frequently found in the 60–69 years age group (47.3%) (Table 1). Typical benign prostate diseases were benign prostate hyperplasia (BPH) (118 cases) with 34.7% being PET-positive. PSA was positive in 83.0% of BPH cases.

**Table 1:** Number of subjects suspected for prostate cancer by screening FDG-PET and/or one of other screening tests, and its positive predictive value

Age group	Number of subjects	Positive case	Found cancer	Detection rate (%)	PPV (%)
30-39	5,073	4	0	0.00	0.0
40-49	15,764	13	0	0.00	0.0
50-59	30,615	102	26	0.08	25.5
60-69	29,382	245	78	0.27	31.8
70-79	9,931	123	54	0.54	43.9
80-	1,490	17	7	0.47	41.2
Total	92,255	504	165	0.18	32.7

PPV: positive predictive value

### FDG-PET (PET scanner and PET/CT scanner)

A significant difference was found in the relative sensitivity between dedicated PET scanner (20.4%) and PET/CT scanner (44.0%) (*P*<0.005). However, FDG-PET was far less sensitive (37.0%) than pelvic MRI (66.5%) and PSA (92.2%) (*P*<0.001). As far as a screening combination is concerned, FDG-PET did not contribute to improving relative sensitivity and PPV (Table 2). Of 154 cases of prostate cancer found by FDG-PET and/or PSA, 33.8% of them were found FDG-PET-positive, and 92.2% PSA-positive. It should be noted that 3.9% of them were FDG-PET-positive and PSA-negative; and they were mostly well-differentiated prostate cancer, but there were no common features among them in terms of staging. As for FDG-PET combined with PSA or pelvic MRI, the relative sensitivity and PPV were 98.8% and 32.8% respectively.

**Table 2:** Detection of prostate cancer by screening FDG-PET and/or by each of the combined screening tests.

Screening test	Number of cases				Performance of each test		Performance if combined with FDG-PET	
	Total	Positive*	Proven cancer*	Unfound cancer*	Relative sensitivity** (%)	PPV** (%)	Relative sensitivity** (%)	PPV** (%)
FDG-PET***	504	186	61	104	37.0	32.8	-	-
Dedicated PET scanner	148	31	10	39	20.4	32.3	-	-
PET/CT scanner	356	157	51	65	44.0	32.5	-	-
Pelvic MRI	227	152	55	29	66.5	36.2	67.9	33.5
Serum PSA	466	387	142	12	92.2	36.7	96.1	34.0

* Out of the cases who underwent the test.

** Analyzed on the subset of cases who underwent the test.

*** FDG-PET includes dedicated PET and PET/CT. PPV: positive predictive value, PSA: prostate specific antigen

All prostate cancers were adenocarcinoma on pathology. Most prostate cancers were classified as Stage II, but FDG-PET could detect only 31.5% of cancers in Stage II, whereas it detected more cases of advanced cancer over Stage III (80.0%) (Table 3).

**Table 3:** Relative sensitivity for prostate cancer of FDG-PET and of other screening test, according to clinical and pathological UICC stage.

Subject	Stage	Sensitivity		
		FDG-PET	Pelvic MRI	PSA
Clinical and Pathological stage	II or less	31.5 (17/54)	62.5 (15/24)	90.4 (47/52)
	III	22.2 (2/9)	100.0 (5/5)	100.0 (8/8)
	IV	80.0 (4/5)	100.0 (2/2)	66.7 (2/3)

UICC: Union for International Cancer Control

Out of the 504 subjects, 116 had undergone the FDG-PET cancer screening program a year before as well with or without the same combined tests. Their final results were 31 cases of cancer, 65 cases of benign disease, and 20 cases that required strict follow-up. The staging results were obtained only for 14 of the 31 proven cases subjects, and 11 cases were in stage II, 1 case was in stage III and 2 cases in stage IV.

## Discussion

FDG-PET has several limitations for the detection of prostate cancer because prostate cancer frequently does not present increased glucose metabolism, and the excretion of FDG into urine often interferes with detection of it (12, 13). The relative sensitivity of FDG-PET in detecting primary prostate cancer was 64%-70.8%, limited to the subjects with high PSA levels, advanced clinical stage, and aggressive cancers (14, 15). These results indicated that FDG-PET contributes little to early prostate cancer detection.

An interesting finding from this survey is the higher sensitivity of PET/CT than of PET. PET/CT, which can obtain functional and anatomical information in a single examination, provides a greater advantage for detecting cancer (16). Therefore the advantage of PET/CT might contribute to improve the sensitivity of prostate cancer detection. Hwang *et al*, reported that 184 of 12,307 patients who underwent PET/CT (1.5%) incidentally showed abnormal hypermetabolism in the prostate. Additional examination was required in approximately 65% (120/184) and prostate cancer was proven in 12.5% (23/184) of PET/CT-positive subjects (0.19% of the total subjects) (17). FDG-PET/CT-positivity indicated presence of cancer with high risk (a Gleason score of 7 or greater), suggesting that a case with abnormal FDG uptake findings should be treated carefully (15, 17). However, there are a significant number of overlapping cases among FDG accumulation in the normal prostate, BPH, and prostate cancer (18, 19).

MRI has been effective for detection of prostate cancer (20). In the National Comprehensive Cancer Network (NCCN) guidelines for the early detection of prostate cancer, multi parametric MRI imaging is regarded as an additional examination aiding the patient with persistent PSA elevation but negative TRUS-guided biopsy (21, 22). In the present survey, pelvic MRI failed to detect stage II or lower prostate cancer. Therefore, FDG-PET combined with pelvic MRI did not exceed PSA sensitivity for prostate cancer screening.

The recent NCCN guidelines selected a PSA value of 1.0 ng/mL for the cut off value of PSA (21) and TRUS-guided biopsy was recommended for cases with PSA 4-10 ng/mL. Therefore further examinations should be recommended for subjects with PSA of 4 ng/mL or greater in this FDG-PET screening program, regardless of the results of other examinations.

One of the limitations of the present survey is that the PSA cut-off value was determined by each facility and was variable. Another limitation of this survey was the inadequate investigation of subjects judged as negative in the FDG-PET cancer-screening program. Moreover, this screening program was performed at the subjects’ request, so the sample may have been biased toward younger subjects for prostate cancer prevalence. In conclusion, the FDG-PET screening program in Japan detected prostate cancer at an early stage, but many of these cancers could be detected by measurement of PSA. PSA should be included in FDG-PET cancer screening programs for prostate cancer.

## Conclusion

The sensitivity of PET/CT scanner was higher than that of dedicated PET. However the sensitivity of FDG-PET was lower than that of PSA and pelvic MRI. Moreover FDG-PET did not contribute to improving the sensitivity and PPV when performed as combined with other screening tests. PSA should be included in FDG-PET cancer screening programs to screen for prostate cancer.
